# The Retinol Binding Protein Receptor 2 (Rbpr2) is required for Photoreceptor Outer Segment Morphogenesis and Visual Function in Zebrafish

**DOI:** 10.1038/s41598-017-16498-9

**Published:** 2017-11-24

**Authors:** Yi Shi, Elisabeth Obert, Bushra Rahman, Bärbel Rohrer, Glenn P. Lobo

**Affiliations:** 1 Department of Medicine, Medical University of South Carolina, Charleston, SC 29425 USA; 2 Tianjin Medical University Eye Hospital, Tianjin, 300384 China; 3 Department of Ophthalmology, Medical University of South Carolina, Charleston, SC 29425 USA; 4 Ralph H. Johnson VA Medical Center, Division of Research, Charleston, SC 2940 USA

## Abstract

Vitamin A (all-*trans* retinol) plays critical roles in mammalian development and vision. Since vitamin A is food-derived, tissue-specific uptake and storage mechanism are needed. In the eye, uptake of RBP4-retinol is mediated by the receptor Stra6, whereas the receptor mediating RBP4 binding and retinol transport into the liver has just recently been discovered. Here we examined the role of zebrafish retinol binding protein receptor 2 (Rbpr2) for RBP4-retinol uptake in developing embryos, using eye development and vision as sensitive readouts. In cultured cells, Rbpr2 localized to membranes and promoted RBP4-retinol uptake. In larvae, Rbpr2 expression was detected in developing intestinal enterocytes and liver hepatocytes. Two *rbpr2* mutant zebrafish lines, each resulting in Rbpr2 deficiency, exhibit a small eye defect, and systemic malformations including hydrocephaly and cardiac edema, phenotypes associated with vitamin A deficiency. In the retina, Rbpr2 loss resulted in shorter photoreceptor outer segments, mislocalization and decrease in visual pigments, decreased expression of retinoic acid-responsive genes and photoreceptor cell loss, overall leading to a reduction of visual function. Together, these results demonstrate that Rbpr2-mediated RBP4-retinol uptake in developing liver and intestine is necessary to provide sufficient substrate for ocular retinoid production required for photoreceptor cell maintenance and visual function.

## Introduction

Vitamin A/all-*trans* retinol/atROL and its metabolites (retinoids) play critical roles in human physiology, including eye development, maintenance of the visual system and photoreception^1–3^. The aldehyde metabolite of vitamin A, 11-*cis* retinaldehyde (11-*cis* RAL), functions as the visual chromophore^3–7^, while the acid form of vitamin A, all-*trans* retinoic acid (RA), regulates gene transcription, by providing ligands for RAR (retinoic acid receptors) and RXR (retinoid X receptors) nuclear receptor transcription factors, influencing genes involved in embryonic eye patterning and photoreceptor development^1,2^. Vitamin A deficiency (VAD) is the third most common nutritional deficiency in the world, affecting development and vision in millions of children and pregnant women^7,8^. A prerequisite for initiation of retinoid-dependent ocular physiological processes is the production of biologically active retinoids from circulating vitamin A precursors^9,10^. Effective distribution of vitamin A throughout the body maintains retinoid signaling in peripheral tissues and ensures photoreceptor function and survival in the eye^11–15^. All-*trans* ROL bound to the retinol binding protein RBP4 (holo-RBP4) serves as the major transport form for vitamin A in the blood^4^. Therefore, molecular mechanisms influencing the uptake of holo-RBP4 for retinoid production play an important role in eye development and in sustaining vision^1,2^.

Retinoids and provitamin A carotenoids are delivered to cells and tissues involving a number of different mechanisms^6,10^. While recent studies in Drosophila have implicated the scavenger receptor class B type I (SR-B1) in cellular uptake of dietary pro-vitamin A carotenoids for all-*trans* retinol production, and we have confirmed that the SR-B1 receptor does indeed facilitate dietary carotenoid absorption in the intestine in the mouse^16–19^, however SR-B1 is not expressed in the liver nor involved in dietary atROL absorption, indicating the presence of other membrane receptors^10,14,20^. Biochemical studies indicate the involvement of a cellular retinol binding protein receptor in vitamin A uptake from circulating holo-RBP4^10^. This receptor was identified as *STRA6* (stimulated by retinoic acid 6) gene product^10,21^. STRA6 is highly expressed in epithelial barriers with tight junctions, including the retinal pigmented epithelium (RPE), the choroid plexus (CP), the blood-brain barrier and Sertoli cells^22,23^. While, it is well established that STRA6 mediates cellular uptake of holo-RBP4 into the eye, STRA6 is not expressed in adult liver nor intestine, the tissues that are proposed to mediate uptake, storage and distribution of all-*trans* retinol in the body, indicating the presence of yet unidentified membrane receptors which could bind RBP4 in this process^20^. The intestinal transport and liver storage of holo-RBP4 might be mediated by the recently identified retinol binding protein receptor 2 (*RBPR2*, also termed as 1300002K09Rik: GeneID: ENSMUSG00000028327)^20^. RBPR2 in mouse is highly expressed in the placenta, adult intestine and liver, but to a lesser extent in the pancreas^20^. *RBPR2* has been classified as a novel receptor for whole body retinoid homeostasis due to its intestinal and liver expression patterns and its ability to bind RBP4^20^. RBPR2 is highly conserved among vertebrates and although the protein structure for RBPR2 has yet to be solved, the encoded protein is proposed to be structurally related to STRA6^20–23^. While loss of function studies of STRA6 have confirmed its importance for eye development, the functional and physiological role of the RBPR2 receptor in retinoid homeostasis for photoreceptor cell maintenance and in the support of vision has not yet been explored in a suitable vertebrate model.

To address this open question, we characterized the function of Rbpr2 in cell culture and established zebrafish models to elucidate the biochemical and developmental consequences of RBPR2 deficiency and used eye development and visual function as end point readouts. Here, we describe the role of Rbpr2 in photoreceptor cell development and visual function, using *rbpr2* mutant zebrafish lines analyzed by histology, immunohistochemistry, optokinetic response tests and transmission electron microscopy (TEM). Using cell culture, we show that zebrafish Rbpr2 is a membrane protein and capable of atROL uptake from its plasma protein bound form (holo-RBP4), activity of which was enhanced in cells co-expressing LRAT. Mutant *rbpr2* zebrafish generated by TALEN-mediated elimination of *rbpr2* showed both abnormal eye development and loss of photoreceptor outer segments. As the zebrafish *rbpr2* mutant was a global knockout, we also observed defects in several organs including the brain (hydrocephalus) and heart (cardiac edema), organs which are heavily dependent on retinoid signaling during development. Loss of Rbpr2 resulted in disrupted photoreceptor outer segments, reduced levels and mis-localization of rhodopsin and cone opsin proteins, down-regulation of key retinoic acid responsive genes and photoreceptor cell death, altogether manifesting in a loss of visual function. Finally, the mutant phenotype was rescued using all-*trans* retinoic acid (ATRA), indicating that sub-optimal levels of the vitamin A metabolite, ATRA, in *rbpr2* mutants results in multi-systemic including severe eye phenotypes. Our observed eye phenotypes and results were consistent with vitamin A deficiency, previously observed in animal models, and suggested that Rbpr2 is required for photoreceptor outer segment morphogenesis and maintenance, likely by contributing to overall retinoid homeostasis.

## Results

### Rbpr2 mediates RBP4-retinol uptake in NIH3T3 cells

**Figure 1:**
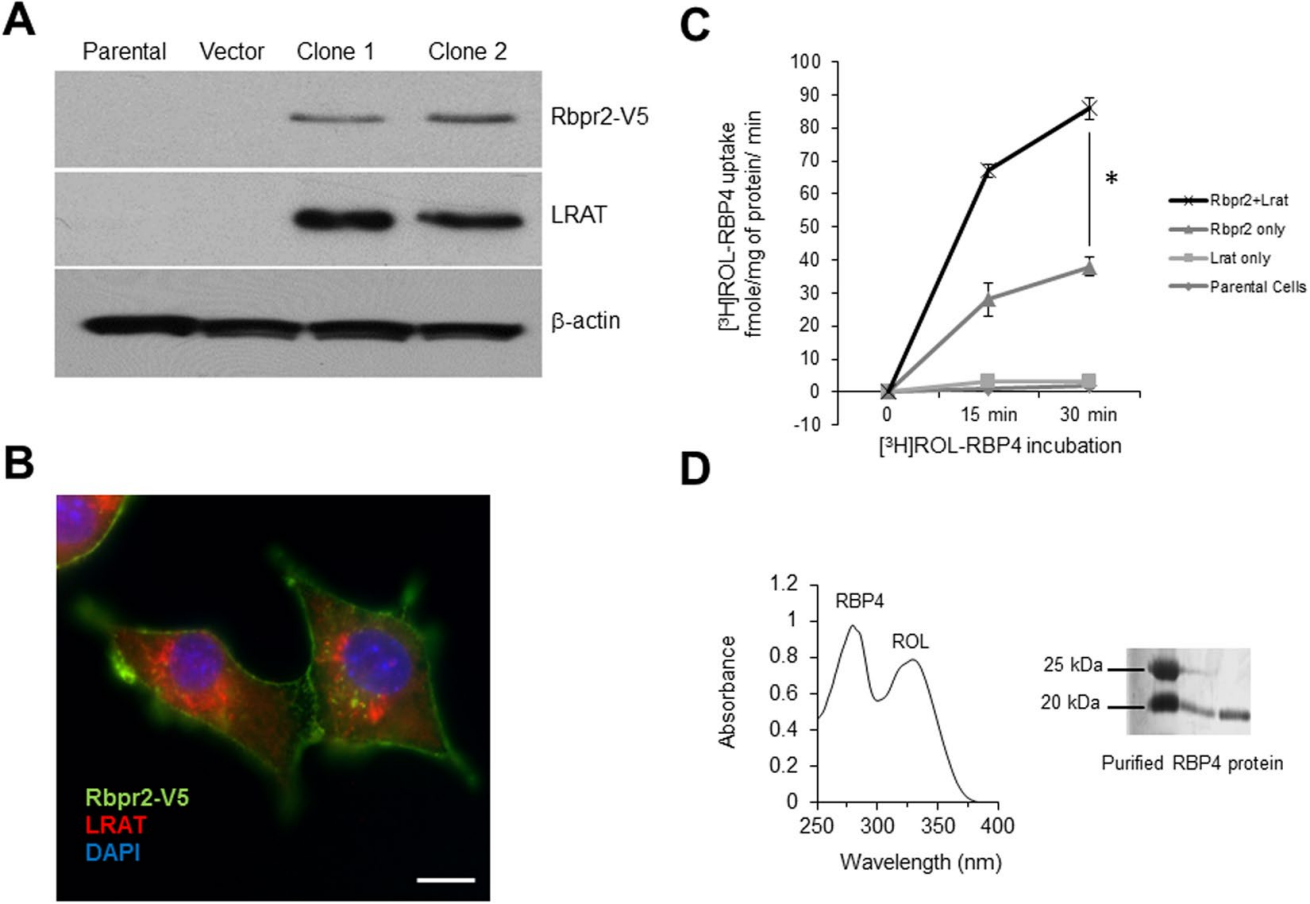
Zebrafish Rbpr2 mediates retinol uptake in NIH3T3 cells. (**A**) Western blot analysis confirmed co-expression of zebrafish Rbpr2 and human LRAT proteins in stable NIH3T3 clones. (**B**) Subcellular localization of zebrafish Rbpr2 (V5-tagged, green) and human LRAT (red) in stable NIH3T3 cells determined by immunohistochemistry and confocal microscopy. Nucleus, stained with DAPI (blue). Scale bar = 50 μm. (**C**) Parental NIH3T3 cells (diamond) or NIH3T3 cells expressing LRAT only (squares) or NIH3T3 cells expressing Rbpr2 only (triangle) or NIH3T3 cells co-expressing Rbpr2+LRAT (X) were incubated with [^3^H]ROL-RBP4. Cells were washed thrice and lyzed at the 0, 15 and 30 min time points. Protein concentrations were estimated and cells were subjected to scintillation counting. The *x*-axis shows the concentration of retinol-bound RBP4 taken up by the cells and expressed as fmole/mg of protein/min. (**D**) Representative images of absorption spectrum of recombinant RBP4-ROL at A280/330 nm. *Inset*, purified recombinant RBP4 protein, resolved by SDS-PAGE and visualized by Commassie Blue staining. *p < 0.005; [^3^H]ROL-RBP4 uptake values in NIH3T3 cells co-expressing Rbpr2 + LRAT compared to NIH3T3 cells expressing Rbpr2 receptor only.

Cellular uptake and transport of lipids, including vitamin A, is proposed to be facilitated by membrane transporters and receptors^4,8–10,20,21^. To confirm that zebrafish Rbpr2 mediates the cellular uptake and transport of RBP4-ROL, stable NIH3T3 cell lines expressing both zebrafish Rbpr2 and human lecithin:retinol acyltransferase (LRAT)^24,25^ were established. Expression of Rbpr2 and LRAT was confirmed by immunoblotting and immunohistochemistry (Fig. 1A). By immunohostochemistry, we observed that V5-tagged Rbpr2 was localized primarily at the plasma membrane and, to some extent, organellar membranes within the cell (Fig. 1B). LRAT on the other hand was localized to the membrane of the endoplasmic reticulum (ER) as shown previously in NIH3T3 cells^24,25^. The localization pattern for zebrafish Rbpr2 was similar to that previously observed for human STRA6, zebrafish Stra6 and mouse RBPR2 in NIH3T3 cells, suggesting conserved functionality for a proposed RBP4-ROL receptor among species^20,21,25^. To determine if Rbpr2 mediates RBP4-ROL uptake, control and different stable cell lines were incubated with [^3^H]ROL-RBP4 and then analyzed for their capability to take up RBP4-ROL by scintillation counting. ROL bound to its plasma protein carrier (RBP4-ROL) was identified in cells expressing Rbpr2, a process that was significantly enhanced in cells expressing both the transporter Rbpr2 and the enzyme LRAT, which catalyzes the transfer of the acyl group from the sn-1 position of phosphatidylcholine to retinol, producing retinyl esters^20,23^ (Fig. 1C,D). Control cells, showed no uptake of RBP4-ROL, suggesting that Rbpr2 could be a bona fide membrane receptor for RBP4 (Fig. 1C). Taken together, these data further warrant the investigation of zebrafish Rbpr2 receptor in the uptake of RBP4-ROL for ocular retinoid production.

### Rbpr2 is expressed in tissues involved in all-trans ROL uptake and storage

**Figure 2:**
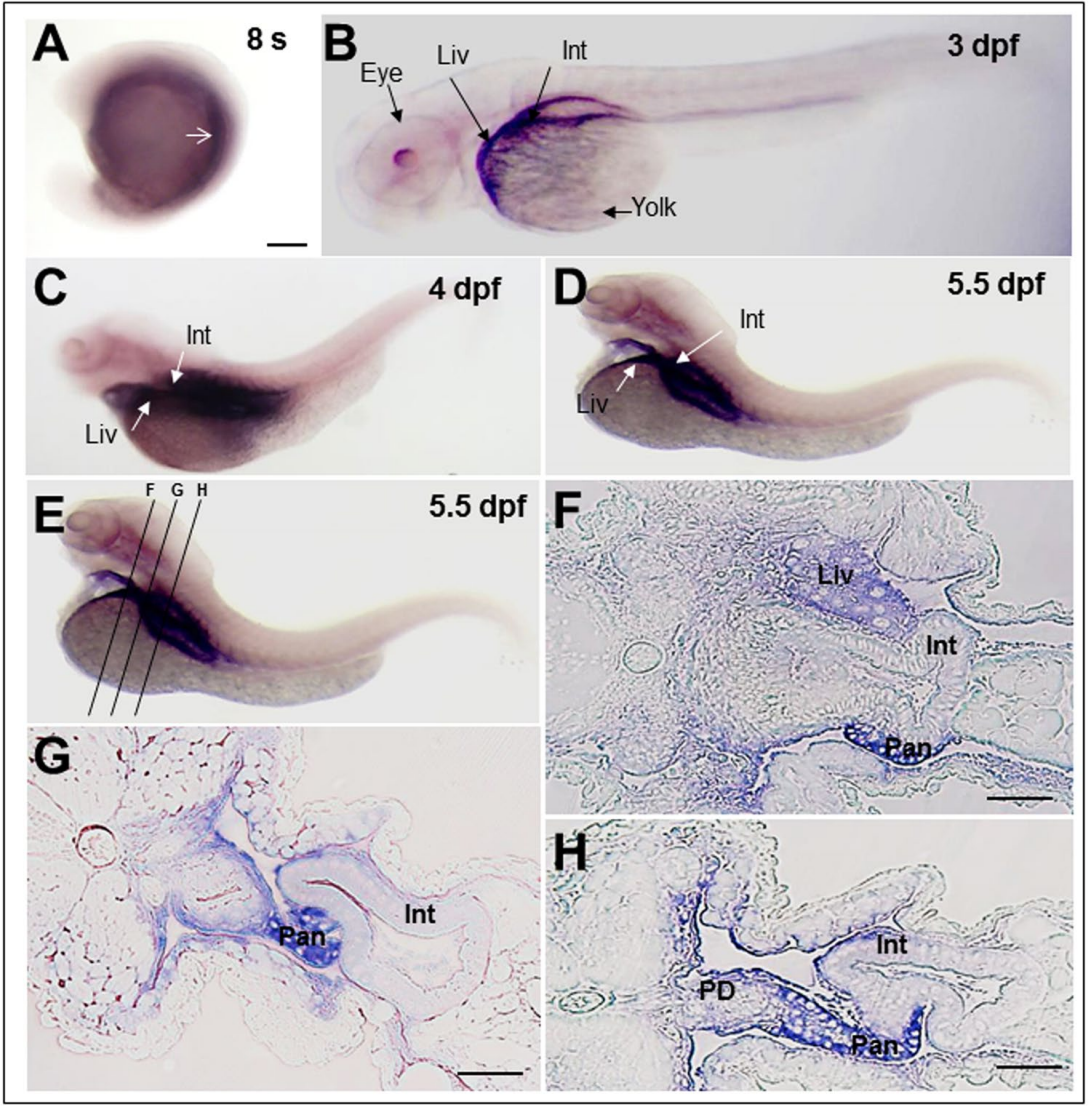
Rbpr2 mRNA expression patterns during zebrafish development analyzed by whole mount *In-situ* Hybridization (WISH). (**A**) At the 8-somite stage, zygotic *rbpr2* mRNA (purple stain) is expressed in the yolk syncytium and in the mesendodermal cells. Staining is also detectable in the anterior somites (arrow). (**B**) At 3 days post fertilization (dpf), staining for *rbpr2* mRNA expression (purple stain) becomes restricted and is observed in the developing liver (Liv) and Gut (Gut). At the 4 (**C**) and 5.5 dpf (**D**) larval stages, *rbpr2* mRNA expression is maintained within the liver and intestine. (**B**–**D**) Interestingly, unlike zebrafish *stra6* mRNA expression in the eye^25^, *rbpr2* mRNA expression was not observed in the developing eyes. (**E**) Transverse sections through 5.5 dpf zebrafish larvae at three different regions, corresponding to panels F–H. (**F**–**H**) Histological analysis of transverse sections reveals *rbpr2* mRNA expression (blue) in the liver hepatocytes (Liv) and intestinal enterocytes (Int). Pan, pancreas; PD, pancreatic duct; Liv, liver; Int, intestine. Scale bar = 100 μm (**F**–**H**).

The developing zebrafish larvae rely on retinoid stores contained in endogenous yolk to be actively transported to the developing eye for patterning and in the support of vision^25–27^. Since most of the all-*trans* ROL metabolizing enzymes, including retinol binding protein Rbp4^28^ have been previously shown to be expressed at early zebrafish embryonic and larval stages, we analyzed if Rbpr2 is also expressed in the developing zebrafish at crucial eye developmental stages^29,30^. To investigate the temporal expression pattern of *rbpr2* in early development, *rbpr2* transcripts were analyzed by semi-quantitative PCR from embryos to 5.5 dpf (days post fertilization) larval stages^29,30^. This analysis revealed that zygotic expression (which begins at 4.5 hours post fertilization in zebrafish^29^) of *rbpr2* transcripts increased from 24 hours post fertilization (hpf) to reach its peak by 4 dpf and was maintained up to 5.5 dpf (latest time point analyzed; (Supplementary Fig. 1)). *Rbpr2* mRNA expression patterns were then analyzed with antisense rbpr2-RNA probes by whole-mount *in situ* hybridization (WISH). WISH analysis showed that *rbpr2 mRNA* is expressed initially in the yolk syncytial layer and in mesendodermal cells during early somitogenesis/segmentation stages (8 somite stages, 8 s), and continuing through larval stages (Fig. 2A–D). Rbpr2 expression was evident in the intestine, liver and pancreas from 3–5.5 days post fertilization ((dpf); 5.5 dpf was the latest time point investigated)) (Fig. 2). Interestingly, unlike zebrafish Stra6, Rbpr2 was not expressed in the larval eye^25^ (Fig. 2B–D), indicating specificity of this RBP4-ROL receptor in tissues associated with RBP4-ROL uptake and storage. To determine specific subcellular distribution patterns of Rbpr2 in these organs, 5.5 dpf WISH stained embryos were embedded in JB-4 plastic resin, and cross sections were collected (Fig. 2E). This analysis showed that Rbpr2 was expressed primarily within the intestinal enterocytes, liver hepatocytes and pancreatic cells (Fig. 2F–H). Because these tissues mediate uptake, storage, and distribution of all-*trans* ROL, the findings suggest that Rbpr2 could play a physiologically relevant role in RBP4-ROL uptake, and likely contributes to overall retinoid homeostasis^20,21^.

### *Rbpr2* mutants show striking gross eye phenotypes normally associated with altered vitamin A metabolism

**Figure 3:**
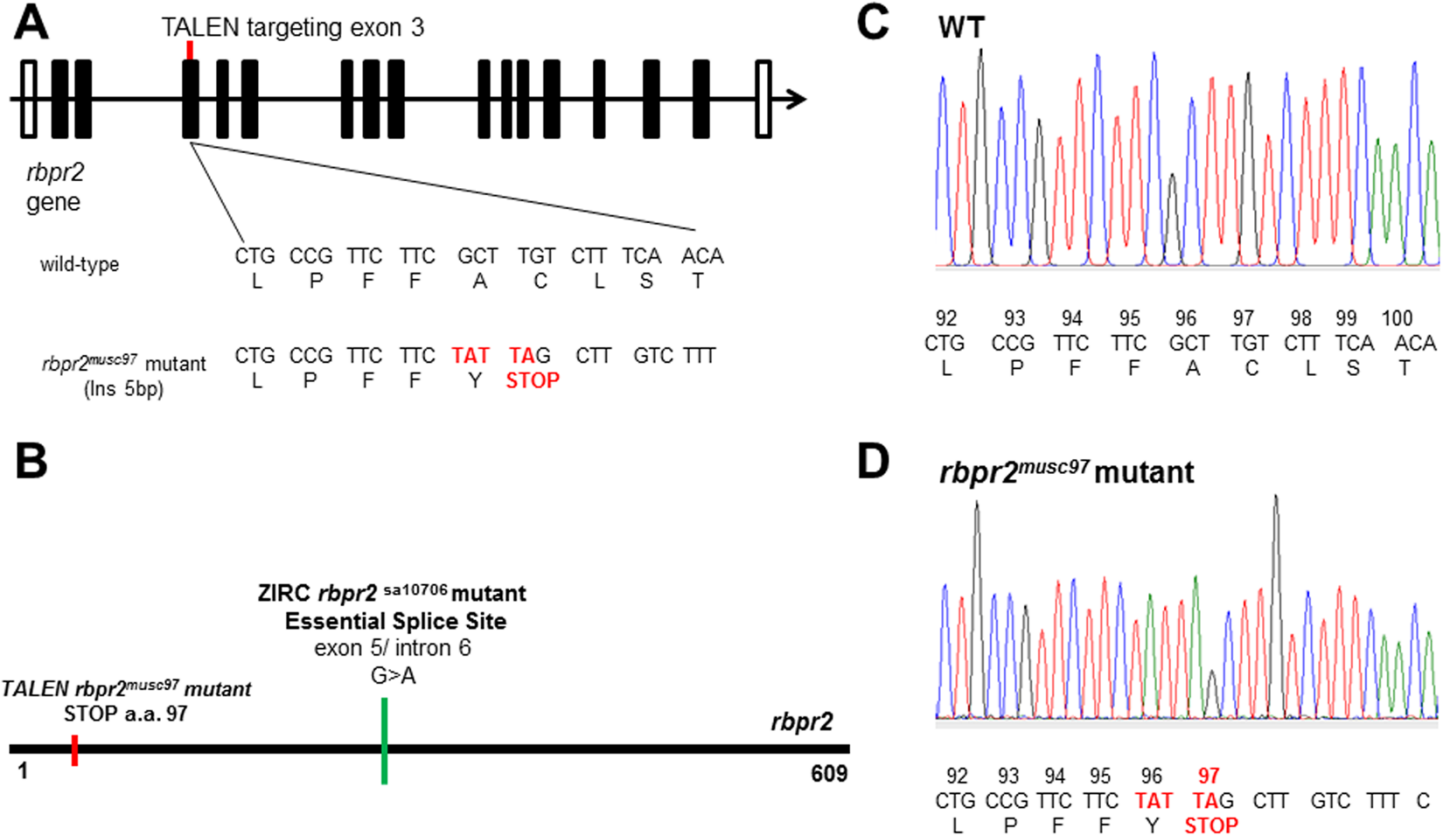
Generation of *rbpr2* mutant (*rbpr2* ^musc97^) zebrafish lines by TALENs. (**A**) Schematic representation of *rbpr2* genomic architecture. The WT sequence and TALENs induced *rbpr2* mutant allele is shown. (**B**) Schematic protein structure of Rbpr2 showing positions of TALEN generated *rbpr2* mutant (*rbpr2* ^musc97^) which results in a premature stop codon at amino acid position 97, and the ZIRC *rbpr2* mutant allele sa10706 (green bar; *rbpr2* ^sa10706^) affecting the essential exon 5/intron 6 splice sites. (**C** and **D**) Chromatograms of Sanger sequencing reactions of WT and homozygous *rbpr2* mutant (*rbpr2* ^musc97^) zebrafish. a.a., amino acid/codon.

**Figure 4:**
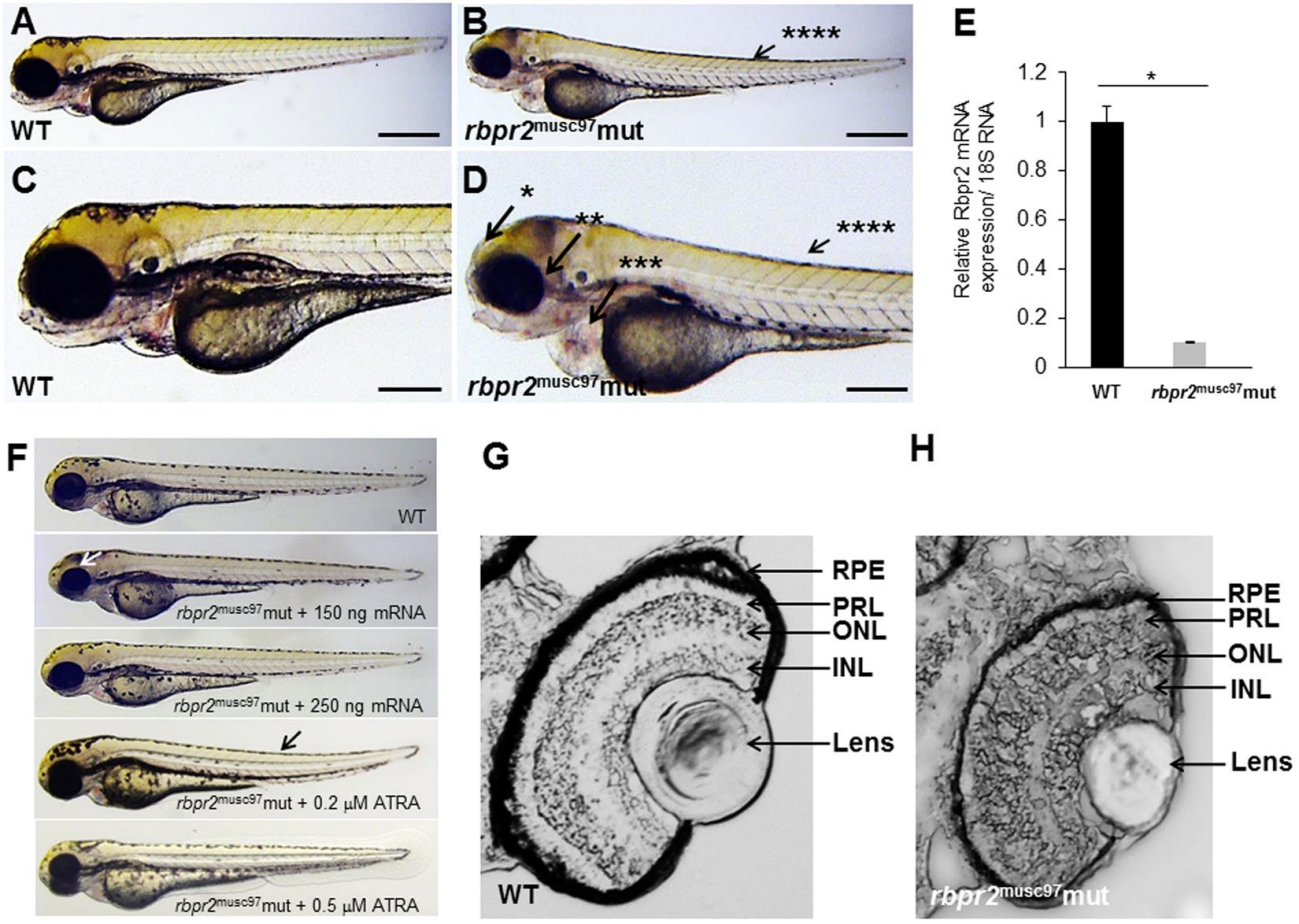
*Rbpr2* ^musc97^ mutants show eye and systemic phenotypes consistent with retinoid deficiency. Lateral view of representative WT (panels A,C) and *rbpr2* homozygous mutant (*rbpr2* ^musc97^ mut, panels C,D) zebrafish at 5.5 dpf. *Rbpr2* ^musc97^ mutants showed gross defects, which included: *hydrocephaly; **smaller eyes, ***pericardial edema and ****slight tail curvature. Scale bars = 0.326 mm (**A**,**B**) and 0.103 mm (**C**,**D**). (**E**) qPCR analysis of *rbpr2* mRNA expression from WT and *rbpr2* ^musc97^ mutant zebrafish larvae at 5.5 dpf. (**F**) Injection of WT zebrafish *rbpr2* mRNA or dose specific treatment with all-*trans* retinoic acid (ATRA) rescues the *rbpr2* ^musc97^ mutant phenotype. Images obtained at 5 dpf. Rescue experiments of *rbpr2* ^musc97^ mutants with either mRNA or ATRA were repeated twice as outlined in methods. (**G**,**H**) Transverse sections of 5.5 dpf WT (panel G) and *rbpr2* ^musc97^ mutant (panel H) eyes. *rbpr2* ^musc97^ homozygous mutant eyes were smaller and show disruption of retinal lamination layers.

In experiments designed to further characterize the physiological role of *rbpr2* in retinal cell development and homeostasis, an *rbpr2* mutant zebrafish line was generated using TALENs^31^. Transcription activator-like effector nucleases (TALENs) were designed against a target in exon 3 of the *rbpr2* gene listed in Ensembl (ENSDARG00000062467) (Fig. 3A,B). The mRNAs encoding the *rbpr2* TALENs were injected into 1 or 2-cell embryos and adult founders were identified by screening offspring for mutations by high-resolution melt analysis to identify variations in nucleic acid sequences (data not shown). *Rbpr2* mutant alleles were subsequently confirmed by sequencing (Fig. 3C,D). The *rbpr2* mutation consisted of a 5 bp insertion, which generated a premature stop codon at amino acid 97 (Fig. 3B–D). The corresponding *rbpr2* mutant zebrafish line (annotated in the manuscript as *rbpr2* ^*musc*97^) was used for all subsequent analyses. By light microscopy, the gross phenotype of *rbpr2* ^*musc*97^ mutants was similar to the Stra6 receptor morphants previously described in zebrafish, with defects in eye size, body curvature and heart formation^25^. At 5.5 dpf, all *rbpr2* ^*musc*97^ mutants showed microphthalamia, pericardial edema and hydrocephalus, features that were never observed in wild-type (WT) or heterozygous siblings (data not shown) (Fig. 4A–D). The morphologic phenotype was consistently observed in 25% of progeny from crosses of heterozygous parents as would be expected for Mendelian inheritance, with *rbpr2* ^*musc*97^ mutants surviving to at least 7–8 dpf. Due to lack of a commercially available anti-Rbpr2 antibody, qRT-PCR analysis was used to confirm that Rbpr2 expression was depleted in *rbpr2* ^*musc*97^ mutant larvae. This analysis showed that Rbpr2 mRNA expression in mutants was significantly depleted (6.62 fold decrease, P < 0.001), as compared to WT controls (Fig. 4E). To confirm that the defects observed in *rbpr2* ^*musc*97^ mutants were caused specifically by loss of Rbpr2, rescue experiments were performed by injecting WT zebrafish *rbpr2* mRNA into control and *rbpr2 mutant* embryos at the 1–2 cell stage. Using light microscopy, at 5 dpf, low dose (150 pg) reconstitution of *rbpr2* mRNA in *rbpr2* ^*musc*97^ mutant embryos partially rescues the eye phenotype (white arrow; Fig. 4F) while high dose (250 pg) *rbpr2* mRNA fully rescues the *rbpr2* ^*musc*97^ mutant phenotype (Fig. 4F). Rescue experiments were also performed with the all-*trans* ROL metabolite, all-*trans* retinoic acid (ATRA). Exogenous applied ATRA was dissolved in DMSO and applied to the fish water containing embryos at the 40% epiboly stage, just before gastrulation begins^30^. Using light microscopy, at 5 dpf low dose ATRA (0.2 μM) treatment resulted in a partial rescue of the mutant phenotype (bent/curve tail phenotype was still present), but a higher dose ATRA (0.5 μM) treatment resulted in a complete rescue of the mutant phenotype (black arrow; Fig. 4F). Histological analysis of *rbpr2* ^musc97^ mutant larvae eyes after both rescue experiments showed normal retinal lamination and eye patterning (Supplementary Fig. 2) which were comparable to WT animals. For controls, embryos were incubated with the vehicle only (0.1% DMSO) in fish water and these showed no rescue of the mutant phenotype at 5 dpf (data not shown). To ensure that the loss of Rbpr2 was causing the gross phenotype, another *rbpr2* mutant from the Zebrafish International Resource Center (ZIRC *rbpr2* ^sa10706^), which affects the essential splice site of *rbpr2* exon5/intron 6 (Fig. 3B), was obtained and analyzed by light microscopy and histology at 5.5 dpf. The resulting phenotype was observed to be similar to the *rbpr2* ^musc97^ mutant phenotype described (Supplementary Fig. 3). Taken together, these data show that loss of functional Rbpr2 in zebrafish leads to gross pathology of the heart, brain and eye, likely due to retinoid deficiency.

### *Rbpr2*^*musc*97^ mutant zebrafish display severe retinal phenotypes

**Figure 5:**
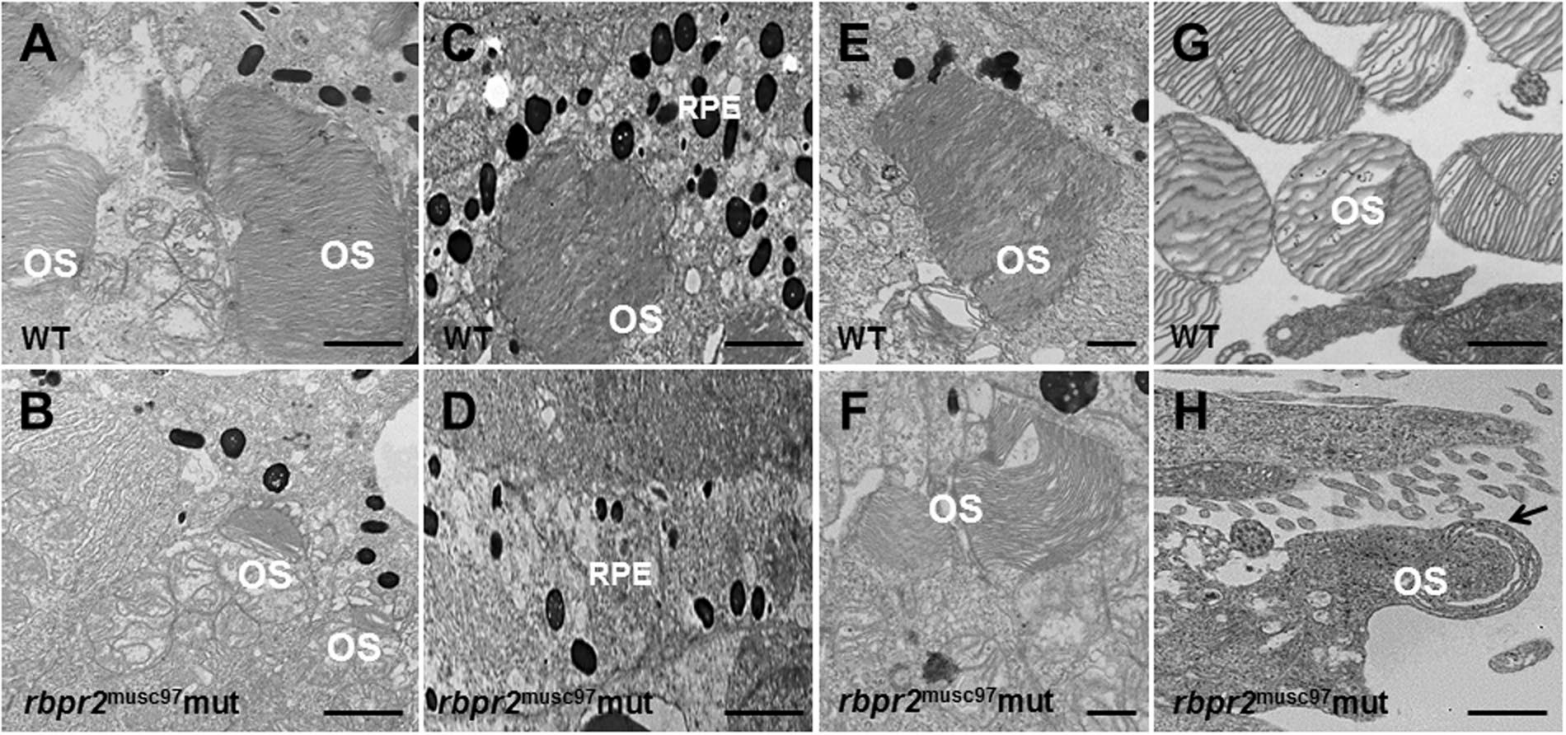
Ultrastructural analysis of WT and *rbpr2* ^musc97^ mutant photoreceptors. Transmission electron microscopy provided ultrastructural views of WT and *rbpr2* ^musc97^ mutant photoreceptor cells at 5.5 dpf. (**A**,**C**,**E** and **G**) WT photoreceptors exhibit tightly stacked outer segment membranes (panels A,C and E; arrows) and RPE cells containing many melanosomes (**C**); (**B**,**D**,**F** and **G**) while in *rbpr2* ^musc97^ mutants only remnants of outer segments (panels B and F; arrows) could be observed, with fewer melanosomes in the RPE cells (**D**). Scale bars = 800 nm (**A**–**E**); 400 nm (**F**); 200 nm (**G** and **H**). OS, outer segments; RPE, Retinal Pigmented Epithelium.

At the 5.5 dpf time point zebrafish visual system is fully developed and the larva is capable of eliciting a visual response to an external stimulus^32^. Therefore, using the 5.5 dpf as the end point analysis, histological analysis, immunostaining and visual function tests were performed on WT and *rbpr2* ^*musc*97^ larvae. In transverse sections, all *rbpr2* ^musc97^ mutants exhibited smaller eyes and disordered retinal cell layers (Fig. 4H v.s. 4G). Interestingly, even as early as 5.5 dpf the outer nuclear layer (ONL) as well as the inner nuclear layer (INL) layer in *rbpr2* ^musc97^ mutants were incompletely formed and appear disorganized, indicating that proper retinal cell development is dependent on functional Rbpr2 (Fig. 4G,H). Disorganization and lack of photoreceptor outer segments (OS) was more obvious by transmission electron microscopy (TEM) of *rbpr2* ^musc97^ mutant larvae at 5.5 dpf. In WT siblings photoreceptor OS contain stacks of ordered membranous discs and extend toward the retina pigmented epithelium (RPE) in a parallel and organized manner (Fig. 5A,C,E). In contrast, the photoreceptor OS in *rbpr2* ^musc97^ mutants were fewer in number; significantly shorter in length and disorganized (Fig. 5B,F). In vertical section disorganized photoreceptor OS, containing discs and whorls of various sizes that lacked the correct orientation, were observed in *rbpr2* ^musc97^ mutants but not WT siblings (Fig. 5G vs. arrow in 5H). Furthermore, the RPE cell layer in *rbpr2* ^*musc*97^mutants was thinner as those seen in WT animals containing fewer melanosomes (Fig. 5D v.s. 5C). Such abnormalities were never observed in retinal sections of WT siblings. Taken together, these results indicate that eye and retinal cell development along with photoreceptor OS development require Rbpr2 function in zebrafish.

### *Rbpr2*^musc97^ mutant zebrafish exhibit cone-rod dystrophy

**Figure 6:**
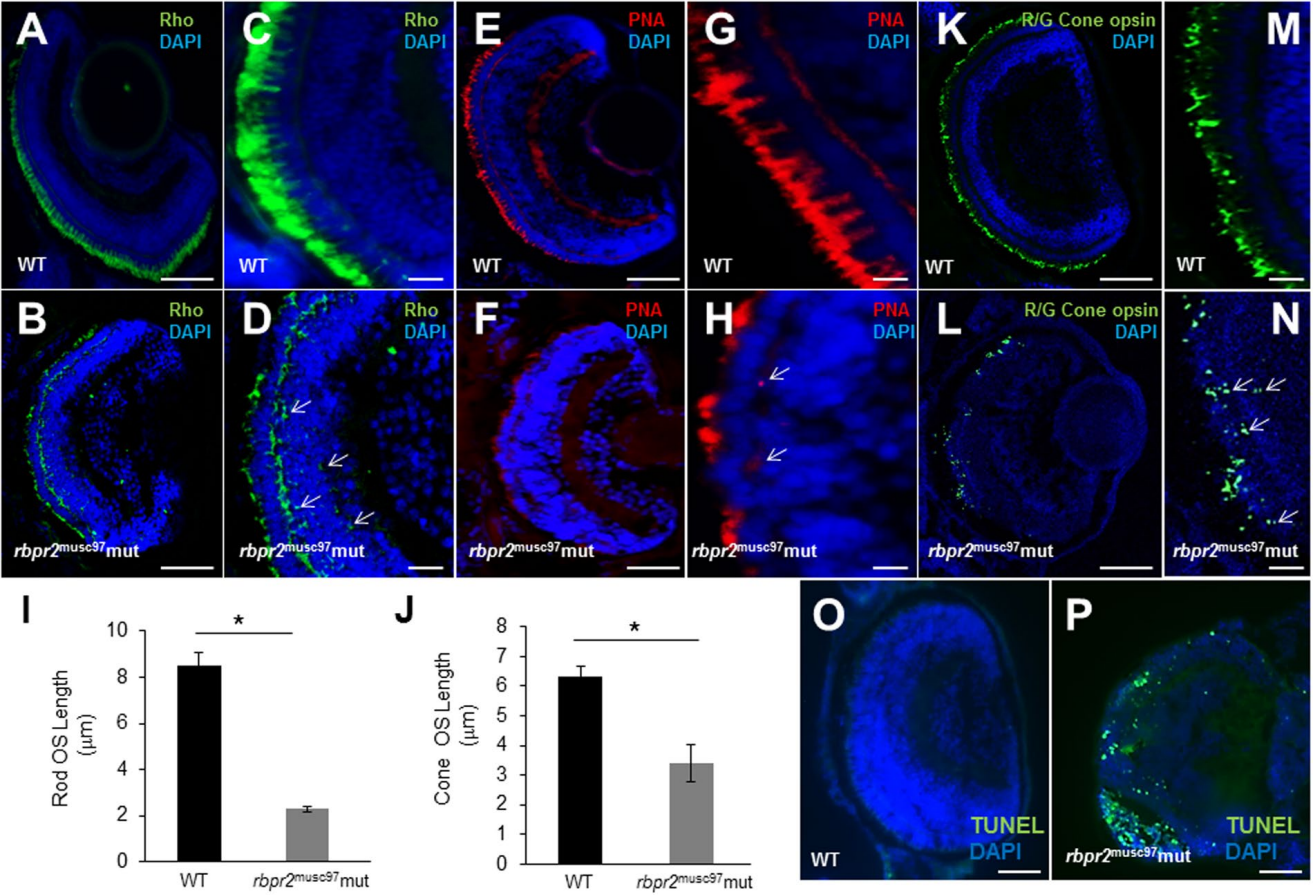
Immunohistochemical analysis of rod and cone photoreceptors in WT and *rbpr2* ^musc97^ mutant zebrafish. Rod photoreceptor outer segments were identified with 1D4 antibody specific for rhodopsin (green, Rho, panels A–D). Cone photoreceptors outer segments were identified with PNA-488 (red, PNA, panels E–H) and Red/Green Opsin antibody (green, R/G Cone opsin, panels K–N) all at 5.5 dpf. Opsin mislocalization was observed in *rbpr2* ^musc97^ mutants (indicated by white arrows in **D**,**H** and **N**). Severe loss of rod and cone pigment proteins was evident in the *rbpr2* ^musc97^ mutant zebrafish (**B**,**D**,**F**,**H**,**L** and **N**). Quantification of photoreceptor outer segment length at 5.5 dpf is provided for rods (**I**) and cones (**J**). TUNEL staining for apoptosis in WT (**O**) and *rbpr2* ^musc97^ mutant (**P**) zebrafish retinas at 6 dpf. TUNEL positive cells/apoptotic nuclei stain green. Scale bars: 100 μm (**A**,**B**,**E**,**F**,**K**,**L**,**O** and **P**); 25 μm (**C**,**D**,**G**,**H**,**M** and **N**).

Since photoreceptor cell abnormalities can result in defective OS protein localization, immunohistochemistry was used to determine if localization of photoreceptor OS proteins occurred normally in *rbpr2* ^musc97^ mutants. At 5.5 dpf, rhodopsin localized normally to the rod OS of WT zebrafish (Fig. 6A,C). In *rbpr2* ^musc97^ mutants rudimentary OS localization of rhodopsin was observed, and rhodopsin was mislocalized to the inner segments (Fig. 6B,D). To quantify the lengths of the OS rhodopsin immunoreactivity was used as a surrogate by determining the extent of rhodopsin staining along the proximal-distal axis of the OS. In WT siblings rod photoreceptor OS were 8.1 ± 0.65 μm in length (*n* = 25 embryos), while *rbpr2* mutant rod OS were 2.2 ± 0.15 μm in length (72% shorter, *P* < 0.001; *n* = 15 embryos) (quantified in Fig. 6I). Cone morphology, the predominant photoreceptor cell type in the zebrafish retina, was next examined by immunolabeling with anti-peanut agglutinin lectin (PNA-488) antibody, which labels the interphotoreceptor matrix surrounding cone OS, and anti-Red/Green cone opsin antibody. Peanut agglutinin lectin and Red/Green cone opsin staining revealed that the *rbpr2* ^musc97^ mutant cone OS were significantly shorter (6.33 ± 0.22 μm in WT vs. 2.8 ± 0.35 μm in mutants; *P* < 0.001) as shown in Fig. 6E,G,K,M v.s. 6F,H,L,N (quantified in Fig. 6J). Additionally, the number of cone OS were significantly fewer in number in *rbpr2* ^musc97^ mutants compared to WT siblings (Fig. 6H,L vs. 6G,K), and mutants exhibit mislocalized opsin in the cone pedicles, which together suggested that loss of Rbpr2 results in defective cone OS morphogenesis (Fig. 6N; arrows indicate mislocalization of cone opsins). Finally, apoptosis is a common feature of photoreceptor cell death in conditions of suboptimal retinoids^33^. We therefore tested for apoptosis in the retinas of *rbpr2* ^musc97^ mutants by Terminal deoxynucleotidyl transferase (TdT) dUTP Nick-End Labeling (TUNEL) assay, designed to detect apoptotic cells that undergo extensive DNA degradation during the late stages of apoptosis^34^. In 5.5 dpf *rbpr2* ^musc97^ mutant zebrafish, the structure of retina was substantially impaired, and apoptotic-positive signals were detected in particular in the outer nuclear layer (ONL) of the central and peripheral retina (Fig. 6P vs. 6O). Taken together, these data demonstrate that Rbpr2 is required for photoreceptor OS maintenance and survival in zebrafish.

### *Rbpr2*^musc97^ mutant zebrafish eyes show downregulation of retinoid regulated genes

**Figure 7:**
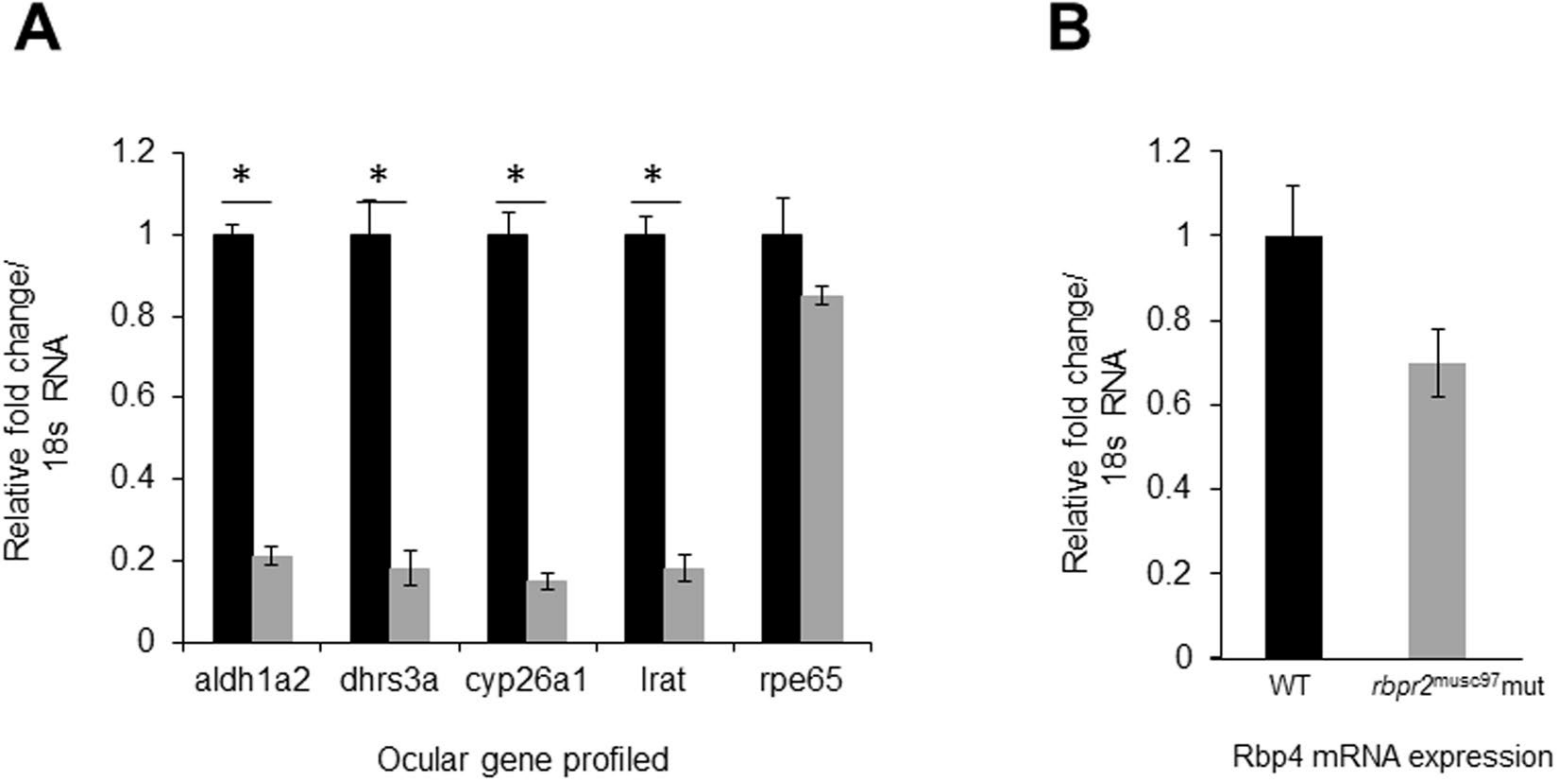
Downregulation of retinoid signaling regulated genes in *rbpr2* ^musc97^ mutants. (**A**) Retina-specific gene expression were compared by qRT-PCR using equal amounts of total RNA from heads of WT (black bars) and *rbpr2* ^musc97^ mutants (grey bars), at 5.5 dpf. *aldh1a2*, *dhrs3a*, *cyp26a1*, *lrat* and *rpe65* mRNA expression were normalized to 18 S ribosomal RNA. mRNA expression values of genes in WT animals were set to 1, and difference in gene expression between the two genotypes are shown as relative fold change normalized to endogenous 18 S RNA. *p < 0.001. (**B**) Whole body Rbp4 expression analysis in WT (black bars) and *rbpr2* ^musc97^ mutants (grey bars) by qRT-PCR analysis.

Gross pathology, retinal histology and rod-cone dystrophy observed in *rbpr2* ^musc97^ mutants, suggested that loss of Rbpr2 likely affects RBP4-ROL uptake resulting in decreased ocular retinoid production. To test this hypothesis, using qPCR mRNA expression of genes containing retinoic acid response elements (RARE) associated with atROL metabolism (retinoid metabolism) and ATRA signaling was measured in *rbpr2* ^musc97^ mutant eyes^33,35–42^. This analysis revealed significantly decreased expression levels of *aldh1a2* (4.8 fold decrease; P < 0.005), encoding a retinaldehyde dehydrogenase that converts retinaldehyde into all-*trans* RA; *dhrs3a* (5.1 fold decrease; P < 0.005), which encodes a dehydrogenase that reduces the amount of retinaldehyde available for conversion to all-*trans* RA, *cyp26a1* (6.3 fold decrease; P < 0.005), which encodes an enzyme that catabolizes all-*trans* RA to non-biological metabolites, and *lrat* (5.8 fold decrease; P < 0.005), the enzyme which produces *all-trans* retinyl esters, in mutants as compared to WT siblings, indicating sub-optimal levels of the ROL and its active retinoid metabolites (Fig. 7; WT, blacks bars; *rbpr2* ^-/-^ mutants, grey bars). Levels of Rpe65, or the retinoid isomerohydrolase, which converts an all-*trans*-retinyl ester to 11-*cis* retinol, were unaffected. Finally, although Rbpr4 mRNA levels were found to be decreased in *rbpr2* ^musc97^ mutants, when compared to controls, differences in Rbp4 expression between these two genotypes did not reach statistical significance (Fig. 7B). Since retinoid production is in part controlled by atROL availability^39,40,42–44^, the quantitative gene expression analysis data strongly suggests that *rbpr2* ^musc97^ mutants have decreased levels of all-*trans* retinol and subsequent metabolites (including ATRA) in the eyes, as compared to WT siblings.

### Visual performance in *rbpr2*^musc97^ mutant animals is significantly compromised

**Figure 8:**
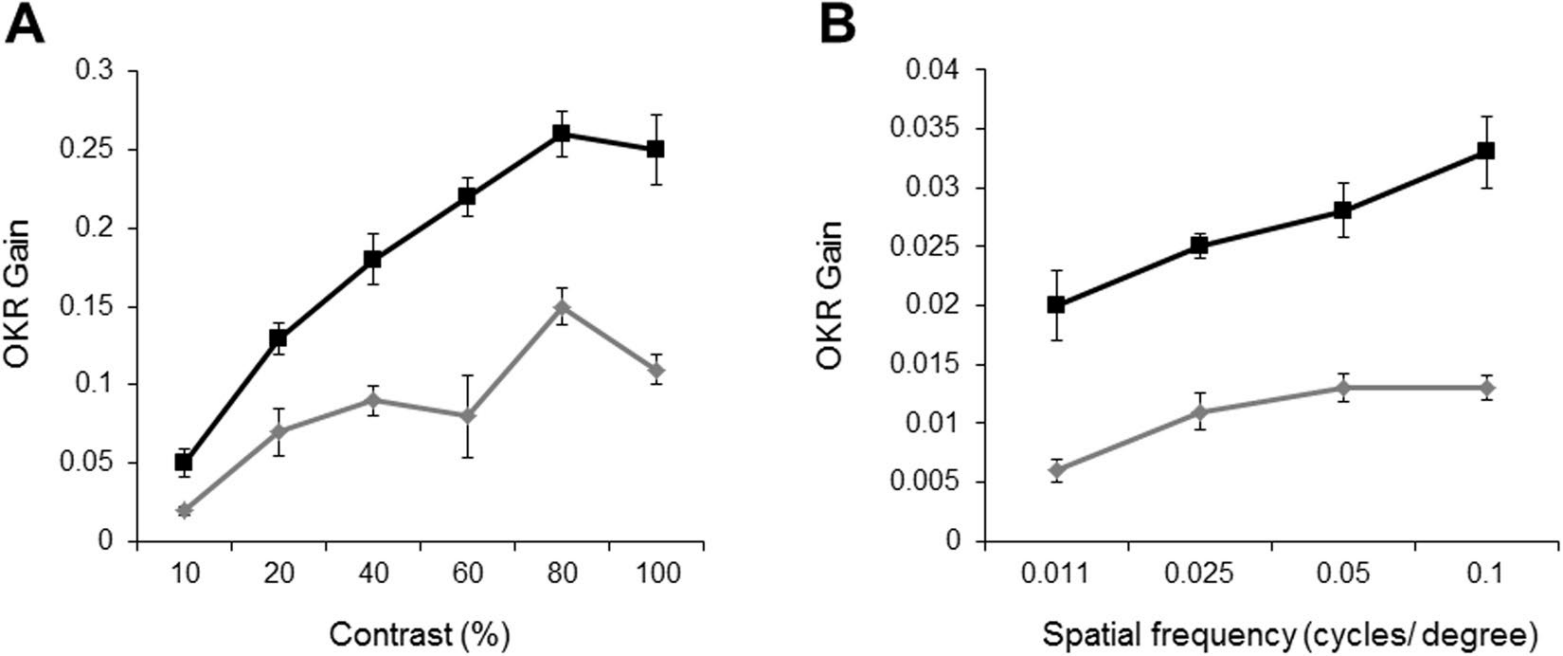
Visual function is affected in *rbpr2* ^musc97^ mutants at 5.5 dpf. Optokinetic response of WT (black squares) versus *rbpr2* ^musc97^ homozygous mutant (grey diamonds) zebrafish at 5.5 dpf as a function of contrast (**A**) and spatial frequency (**B**) measured from smooth pursuit eye movements. Contrast sensitivity test *n* = 16 (mutants) and *n* = 26 (controls). Spatial frequency test *n* = 16 per genotype. Error bars: ± SEM.

It is known that zebrafish visual function is cone-driven at 5.5 dpf^3,32,38^ and since cone OS were disrupted in *rbpr2* ^musc97^ mutant retinas, we hypothesized that visual performance of *rbpr2* ^musc97^ mutants would likely be compromised. We therefore evaluated visual function in control and *rbpr2* ^musc97^ mutants at 5.5 dpf by assessing the optokinetic response (OKR) gain, using the VisioTracker system^32^. The OKR gain is defined as the ratio between stimulus velocity and eye velocity, and is dependent on angular stimulus velocity, spatial frequency, and contrast of the moving image^32^. Reduced gain is indicative of defective visual performance. WT siblings (black squares, Fig. 8A) showed a linear relationship between gain and contrast from 0–80% contrast and between gain and spatial frequency across the entire range tested (0.011, 0.025, 0.05 and 0.1 cycles/degree). Optokinetic response gain in *rbpr2* ^musc97^ mutant animals (grey diamonds, Fig. 8A,B) was significantly compromised when compared to controls in response to varying either the stimulus contrast or spatial frequency. Taken together our histological and functional data revealed a rod-cone dystrophy in *rbpr2* ^musc97^ mutant animals caused by sub-optimal levels of ocular retinoids.

## Discussion

The importance of proper vitamin A levels to embryonic development first became apparent from studies beginning in the 1940s by Warkany and colleagues, who demonstrated that vitamin A deficiency (VAD) results in pleiotropic embryonic defects in multiple organs, including the eyes and heart^45,46^. In fact, for more than 60 years, VAD has been known to cause night blindness and progressive retinal degeneration^2–6^. These early observations are not surprising, as the eye is the human organ most sensitive to vitamin A/Retinol (atROL) deficiency because of vision’s absolute dependence on ocular retinoid availability for embryonic eye patterning, photoreceptor cell maintenance and light perception^1–6^. In light of these observations, it is now known that inadequate vitamin A nutrition during early pregnancy accounts for some pediatric congenital abnormalities, largely affecting eye development, primitive heart development and specification of the hindbrain^8,37^. Vertebrates are, however, unable to synthesize vitamin A *de novo* and must obtain vitamin A from the diet via the intestine, or in case of the developing fetus from maternal sources via the placenta^10,12,37^. Therefore, mechanisms/membrane receptors influencing the uptake of vitamin A in these organs, and for ocular retinoid production, play a significant and direct role in vertebrate eye development and visual function.

Recently, the mouse retinol binding protein receptor 2 (RBPR2) was implicated in the process of vitamin A uptake from its protein bound form (RBP4-ROL), in tissues that do not express STRA6 the only known receptor for holo-RBP4. However, due to the lack of testing in a suitable *in-vivo* model, the physiological role of this membrane receptor in the uptake of RBP4-ROL for retinoid homeostasis and in the support of vision had not yet been established^20^. Using a mammalian cell line stably expressing Rbpr2, we observed that the zebrafish Rbpr2 receptor was membrane localized and capable of RBP4-ROL uptake, and that RBP4-ROL uptake was enhanced in cells co-expressing LRAT in a time dependent manner. Our results were consistent with those observed for mouse RBPR2 and human STRA6, membrane receptors for RBP4-ROL uptake and transport previously analyzed in NIH3T3 cells^20,25^. Next, we generated *rbpr2* mutant zebrafish lines (*rbpr2* ^musc97^) using TALEN technology^31^. Using this mutant *rbpr2* line we studied the consequences of its loss of function on retinoid homeostasis for vertebrate eye development, in photoreceptor cell maintenance and for vision.

We cloned zebrafish *rbpr2* gene and showed that its expression was initiated at early segmentation stages (11–12 hpf) which coincides with the development period (11.5 hpf) of the embryonic optic vesicle^30^. This early expression pattern of Rbpr2 in zebrafish was not surprising as many of the vitamin A metabolizing enzymes including retinol binding protein 4 (Rbp4) are already expressed during gastrulation and somitogenesis stages in zebrafish embryos^28^. At 5.5 dpf *rbpr2* ^musc97^ mutants exhibited severe retinal dystrophy including disruption of retinal lamination layers, shorter and disorganized rod and cone photoreceptors, mislocalized rod and cone opsin, downregulation of retinoic acid (RA) responsive genes and showed significantly reduced visual function, phenotypes previously associated with defective ocular retinoid homeostasis^23,25–27^. Since at later larval stages *rbpr2* mRNA expression was restricted to the developing intestine, liver and pancreas, it suggests that the organism also utilizes this mechanism to acquire dietary vitamin A, however this remains to be tested in mammalian models.

Eye phenotypes of the *rbpr2* ^musc97^ mutant animals were comparable to those previously observed in ocular retinoid depleted mice and zebrafish^23,25–27,43,47–55^. Additionally, our TALEN generated *rbpr2* ^musc97^ mutant zebrafish line phenocopied a previous model of ocular VAD established in zebrafish^25^ and a commercially available *rbpr2* mutant line (ZIRC *rbpr2* ^sa10706^); thus implicating an essential role for the Rbpr2 membrane receptor in retinoid homeostasis in the support of eye development and visual function in vertebrates. The hypothesis that loss of *rbpr2* affects RBP4-ROL binding and uptake invariably diminishing systemic and ocular retinoid content was tested by performing rescue experiments with all-*trans* RA (ATRA) and by measuring expression of ocular genes associated with vitamin A metabolism (retinoid metabolism) and ATRA signaling^35,36,40–42^. ATRA treatment of *rbpr2* ^musc97^mutant zebrafish rescued the mutant phenotype in a dose dependent manner indicating that decreased levels of the all-*trans* ROL metabolite ATRA affects cellular signaling in the mutants. Additionally, qRTPCR analysis for retinoid signaling in the heads of *rbpr2* ^musc97^ mutant zebrafish showed significant down regulation of genes encoding enzymes involved in retinaldehyde to RA conversion (*aldh1a2, dhrs3a*), enzymes that catabolize all-*trans* RA to non-polar metabolite’s (*cyp26a1*), in eyes of *rbpr2* ^musc97^ mutants. Evidence for developmental ATRA deficiency of the eyes, leading to downregulation of retinoic acid responsive genes has been observed in other VAD deficient animal models^23,25–27,43,47–55^. In line with this observation, the malformations in RPE and photoreceptors observed in *rbpr2* ^musc97^ animals are consistent with the known role of ATRA in the patterning of the retina and RPE. Additionally, evidence from RPE cell cultures further suggests an important role of ATRA in the regulation of RPE differentiation, proliferation and melanogenesis^53,54^. Collectively taken our results are consistent with ATRA regulating expression of ATRA metabolic enzymes in an attempt to achieve ATRA homeostasis^43,47–55^, thereby verifying the proposed function of Rbpr2 as a RBP4-ROL transporter for ocular retinoid production required for establishment of the visual apparatus and in the support of vision, in an animal model.

Optimal levels of the ligand (11-*cis* retinal) is required during cone opsin synthesis for successful opsin trafficking and that without 11-*cis* retinal, cones may degenerate because of opsin mislocalization^43,48–55^. In line with this observation, the observed mis-localization of cone opsins (Fig. 6L) suggested that 11-*cis* retinal levels in *rbpr2* ^musc97^ mutant zebrafish are likely diminished a hypothesis that has not yet been tested. Finally, detection of TUNEL positive nuclei in *rbpr2* ^musc97^ mutant zebrafish photoreceptor cells, also suggested that cell death occurred likely due to sub-optimal ocular retinoid levels, establishing the importance of ROL uptake and transport to the eye for retinoid production in photoreceptor OS maintenance and for vision^43,48–55^.

In summary, our study validates and extends the work of Alapatt and colleagues^20^. We show in an animal model that Rbpr2 is a novel RBP4-ROL binding receptor, and plays a functional role in ocular retinoid homeostasis. In cell culture, we demonstrate that the Rbpr2 retinoid channel takes up ROL in an RBP4-dependent manner. In this process, LRAT enhances the RBP4-ROL uptake capabilities of Rbpr2, confirming the functional coupling of LRAT and its respective vitamin A transporters in regulating the binding uptake of holo-RBP4. Furthermore, we provide evidence that Rbpr2 deficiency leads to developmental abnormalities, including severe eye malformations, heart edema and hydrocephaly, pathological conditions previously associated with VAD. Rbpr2 deletion resulted in shorter photoreceptor outer segments, decrease in rod and cone opsin proteins, downregulation of retinoid signaling ocular genes, photoreceptor cell death and a significant decrease in visual function. Thus, our findings in cell culture and zebrafish as presented here require further testing in mammalian models. The vertebrate model presented here, may also serve as a tool, to study mechanisms associated with rod-cone dystrophies, dependent on ocular retinoid signaling.

## Materials and Experimental Procedures

### Materials

All chemicals, unless stated otherwise, were purchased from Sigma-Aldrich (St. Louis, MO, USA) and were of molecular or cell culture grade quality.

### Cloning of the Zebrafish Rbpr2 cDNA

Total RNA (~1 μg) from 3 dpf zebrafish was reverse transcribed using the SuperScript One-Step RT-PCR for LongTemplates system (Invitrogen, Grand Island, NY). The full-length *Rbpr2* cDNA was amplified by using the Rbpr2 forward primer (5′-ATGTTTCTGCTCTCATTAGTGCAGCGGCGa-3′) and the Rbpr2 reverse primer (5′-TCAGATGTCTAGCGGTGCTGGTTCTGTCTCAGC-3′) with the Expand High Fidelity PCR system (Roche, Indianapolis, IN, USA). The amplified *Rbpr2* cDNA product was cloned in frame into the pCDNA3.1 V5/His TOPO vector (Invitrogen, Carlsbad, CA). Appropriate construction of the wild-type *Rbpr2* plasmid in the pCDNA 3.1 V5/His TOPO vector (pRbpr2-V5) was verified by sequence analysis of both strands (Genomics Core Cleveland Clinic Foundation, Cleveland, OH, USA) and by comparing the sequences to the reference zebrafish *Rbpr2/Stra6l* cDNA sequences deposited in Ensembl (www.ensembel.org) and the ZFIN database (http://zfin.org).

### Generation of stable cell lines expressing Rbpr2 or Rbpr2/Lrat

Mouse NIH3T3 cells obtained from American Type Tissue Culture (ATCC-1658) were maintained in high-glucose DMEM supplemented with 10% FBS and 1% penicillin-streptomycin sulfate, and cultured at 37 °C with 5% CO_2_. To generate constitutively expressing zebrafish Rbpr2 in NIH3T3 cells, parental NIH3T3 or NIH3T3/LRAT^25^ expressing cells were transiently transfected with the pRbpr2-V5 plasmid, as described previously^17,18^. 40 h post transfection, media was replaced to contain 400 μg/mL Geneticin (G418) selection agent. After one to two weeks of selection with G418, surviving individual cells (n = 10) were selected by placing cloning rings around each surviving cell. Each cell was then carefully detached by adding 10 μL of trypsin into each cloning ring. Detached cells were transferred to 12-well culture plates containing 200 μg/mL G418 selection media. Once individual clones reached ~90% confluency they were expanded into 100 mm dishes containing 200 μg/mL of G418 selection media. To confirm stable integration of the *rbpr2* gene and expression in these cells, we isolated total protein from each clone and subjected them to western blot analysis. By using the V5-primary antibody (SIGMA) we detected the V5-tagged RBPR2 protein.

### Indirect immunofluorescence and confocal microscopy

Stable cell lines were grown on coverslips and fixed in a freshly prepared mixture of 4% paraformaldehyde in 1X PBS (137 mM NaCl, 2.7 mM KCl, 10 mM sodium phosphate dibasic, and 2 mM potassium phosphate monobasic, pH 7.4) for 30 min at room temperature and processed as previously described^17,18^. Subcellular localization of the recombinant zebrafish Rbpr2-V5 or LRAT in NIH3T3 cells was achieved by exposure to the anti-V5 primary antibody (which detects the V5-tagged Rbpr2) or anti-LRAT (Abcam) followed by the anti-mouse conjugated Alexa 488 or anti-rabbit conjugated 594 secondary antibody staining (Invitrogen, Carlsbad, CA). Cells were examined under a Zeiss LSM 510 UV Meta confocal microscope with an HCX Plan × 40 numerical aperture 1.4 oil immersion objective lens (Zeiss, Jena, Germany). Images were acquired with the Zeiss confocal software, version 2.0. All experiments were carried out in triplicate. Approximately 50–70 cells from 4–5 fields were imaged/counted per experiment.

### Expression and Purification of Human Serum RBP4

RBP4 expression and purification from *Escherichia coli* was accomplished essentially as described previously and performed in the laboratory of Dr. Johannes von Lintig with assistance from Dr. Marcin Golczak (Department of Pharmacology, Case Western Reserve University)^20,25,56^. Briefly, human RBP4 (hRBP4) cDNA was cloned into a pET3a expression vector and expressed in BL-21 DE3 cells according to a standard protocol. Bacterial cells were harvested and lysed by osmotic shock. Insoluble material was pelleted by centrifugation, washed, and solubilized in 7 M guanidine hydrochloride and 10 mM dithiothreitol. After overnight incubation, insoluble material was removed by ultracentrifugation, and the supernatant was used for the hRBP refolding procedure. hRBP was refolded by the dropwise addition of solubilized material into a mixture containing 150 μCi of [11,12-^3^H]ROL ([^3^H]ROL) (PerkinElmer Life Sciences) and non-radiolabeled ROL (Sigma) at a final concentration of 1 mM. Refolded holo-hRBP was dialyzed against 10 mm Tris/HCl buffer, pH 8.0, and loaded onto a DE53 anion exchange chromatography column (Whatman, Piscataway, NJ). Holo-hRBP was eluted with linear gradient of NaCl (0–1 M) in 10 mm Tris/HCl buffer, pH 8.0. Collected fractions were examined by SDS-PAGE and UV-visible spectroscopy to ensure a proper protein/ROL ratio. This protocol typically yields holo-RBP4 at a ratio of 1:0.8^20,25,44^. Fractions were pooled together and concentrated in a Centricon centrifugal filter device (cut-off 10,000 Da) (Millipore, Billerica, MA). [^3^H]ROL-RBP4 was quantified in a scintillation counter (Beckman Coulter, Indianapolis, IN). The quality of the [^3^H]ROL-bound RBP4 complex ([^3^H]ROL-RBP4) was confirmed by reassessing absorbance ratio at A280/330 nm and fractions of 1:0.8 ratio or higher were pooled, concentrated, and stored at −80 °C until further use.

### *In-vitro* RBP4-ROL binding and uptake studies

Stable NIH3T3 cells expressing either, Rbpr2, LRAT or co-expressing both Rbpr2 and LRAT were plated in 10 cm dishes. Cells were grown to 70% confluency, washed thrice with 1x PBS and incubated for 1 h in serum-free medium, at which point 50 nm [^3^H]ROL-RBP4 was added. At 0, 15 and 30 min time points cells were collected, washed thrice with 1x PBS and lysed in PBS containing 1% Nonidet P-40. Lysates were homogenized and transferred to scintillation tubes for scintillation counting. Protein was estimated using the BCA assay. Parental NIH3T3 and NIH3T3-LRAT only expressing cells incubated with [^3^H]ROL-RBP4 served as controls.

### Animal approval

All experiments on zebrafish were approved by the Institutional Animal Care and Use Committee (IACUC) of the Medical University of South Carolina and the Cleveland Clinic, and were performed in compliance with the ARVO Statement for the Use of Animals in Ophthalmic and Vision Research. The *rbpr2* mutant zebrafish line (*rbpr2* ^sa10706^) was obtained from the Zebrafish International Resource Center (ZIRC).

### Zebrafish strains and maintenance

Zebrafish (strain AB/TL) were bred and maintained under standard conditions at 28.5 °C. Collected embryos were maintained in embryo medium (15 mM NaCl, 0.5 mM KCl, 1 mM CaCl2, 1 mM MgSO4, 0.15 mM KH2PO4, 0.05 mM NH2PO4, 0.7 mM NaHCO3) at 28.5 ^0^C. For whole mount *in situ* hybridization experiments, at 12 hpf embryos were raised in embryo medium containing 0.003% 1-phenyl-2-thiourea (PTU, Sigma-Aldrich, St. Louis, MO) to inhibit melanin pigment formation and staged by morphological criteria^30^. Morphological features were used to determine the stage of the embryos in hours (hpf) or days (dpf) post fertilization^30^.

### Semi-quantitative PCR

Total RNA was isolated from staged zebrafish^29,30^ and first-strand cDNA synthesis was achieved by using the High Capacity RNA-to-cDNA kit following the manufacturer’s instructions (ThermoFisher) and as described before^57^. Semi-quantitative PCR was performed with primers as follows: zebrafish (zf) *rbpr2*, 5′-AGGCGGTACGCATCCATCTG -3′ and 5′-ATCTGTAGTCTGCCAGTGTC-3′; zebrafish *stra6*, 5′-GGTCCATGGTCCTGCACAGG-3′ and 5′-CAATCTTCAGGTAATGGGAG-3′; and zebrafish *gapdh* 5′-GATTACATGGTTTACATGTT-3′ and 5′-CAAAGGAGCCAGGCAGTTGG-3′. The PCR program included initial denaturing at 94 °C for 5 min, followed by 25 cycles of denaturation at 94 °C for 30 s, annealing at 58 °C for 30 s, and elongation at 72 °C for 60 s. PCR products were separated by electrophoresis on 2% (w/v) agarose gels and visualized by ethidium bromide staining.

### Whole-mount *in situ* hybridization in Zebrafish

Whole-mount *in situ* hybridization (WISH) in staged zebrafish larvae was performed according to published protocols and as previously described^32,44^. *Danio rerio* rbpR2 cDNA (ZGC: 162946) was cloned into the vector pCRII-TOPO (Invitrogen, Grand Island, NY), and antisense RNA probes were synthesized using T7 polymerase following protocols as outlined by the manufacturer (Roche Applied Sciences, Indianapolis, IN).

### Zebrafish larvae sectioning and imaging

WISH stained embryos for sectioning were first re-fixed in 4% paraformaldehyde and then dehydrated through a standard ethanol series to 100%. Embryos were placed in plastic molds and embedded in either Technovit7100 resin (Heraeus Kulzer, Germany) or Poly/Bed 812 embedding medium (PolySciences, Warrington, PA) as per the manufacturer’s instructions. Hardened plastic blocks containing zebrafish samples were then transverse sectioned (1 μm thickness) using a Leica Rotary Microtome LM2255 (Leica, Germany) with a diamond blade.

### Zebrafish Immunohistochemistry and Fluorescence Imaging

5–5.5 dpf WT or mutant zebrafish (*rbpr2* ^musc97^ or *rbpr2* ^sa10706^) larvae were fixed in 4% paraformaldehyde buffered with 1X PBS for 3 hours at RT. All samples were then cryoprotected in 30% sucrose for 48 hours. Cryosections (10 μM) were cut and dried onto frost-free slides at RT overnight. Blocking solution (1% BSA, 5% normal goat serum, 0.2% Triton-X-100, 0.1% Tween-20 in 1X PBS) was applied for 2 hours in a humidified chamber. Primary antibodies were diluted in blocking solution as follows: anti-1D4/Rhodopsin (1:250, Abcam, Cambridge, MA), anti-lectin PNA-488 (1:2000, Molecular Probes, Eugene, OR), anti-Red/Green cone opsins (1:1000; Millipore St. Louis, MO), and 4′,6-diamidino-2-phenylendole (DAPI; 1:5000) was used to label nuclei. All secondary antibodies were used at 1:3000 concentrations (Molecular Probes, Eugene, OR). Optical sections were obtained with a Leica SP8 confocal microscope (Leica, Germany) and processed with the Leica Viewer software.

### Generation of *rbpr2* mutant zebrafish using TALENs

Transcription activator-like effector nucleases targeting zebrafish *rbpr2* gene were designed with TALENT software (available in the public domain at https://talent.cac.cornell.edu/TALENT/). The Golden Gate assembly method was used to generate the TALEN constructs^31^. We synthesized 5′-capped mRNA encoding the TALENs using the Sp6 mMESSAGE mMACHINE Kit (Ambion; Thermo Fisher Scientific, Waltham, MA, USA) and microinjected 100 pg of mRNA into WT zebrafish embryos at the one-cell stage. To confirm the mutation, genomic DNA from clipped fins, or whole 4 dpf zebrafish with phenotypes, was extracted in 50 μL 1x lysis buffer (10 mM Tris-HCl pH 8.0, 50 mM KCl, 0.3% Tween 20, 0.3% NP40), denatured at 98^o^C for 10 minutes, digested at 55^o^C for 6 hours after 4 μL of 10 mg/mL proteinase K was added, and the reaction was stopped at 98^o^C for 10 minutes. The PCR primers used were: forward primer, 5′-CCATTGTGTACCTGATAGGA-3′ with reverse primer, 5′-CACATGAGCGTGTAGAGAAG-3′. Sequencing was performed by Eurofins with the forward primer, 5′-CCATTGTGTACCTGATAGGA-3′ (Eurofins.com). The ZIRC *rbpr2* sa10706 mutant zebrafish line (*rbpr2* ^sa10706^) which contains a G > A base substitution at the exon5/intron6 splice site, was confirmed by PCR and direct sequencing of the using forward primer, 5-GGTCCTTATGAGAAACCGATCA-3′ and reverse primer, 5-CCCCTACTGAACCTTATTGTACATTTT-3′. Sequencing was performed by Eurofins with the forward primer, 5-GGTCCTTATGAGAAACCGATCA-3′ (Eurofins.com).

### *Rbpr2*^musc97^ mutant zebrafish mRNA rescue experiments

For rescue experiments of zebrafish *rbpr2* mutants (*rbpr2* ^musc97^), capped and polyadenylated mRNA of WT zebrafish *rbpr2* was synthesized *in vitro* using the mMESSAGE mMACHINE kit (Ambion, Austin, TX) and as previously described^58^. Two doses of WT *rbpr2* mRNA, (low: 150 pg) or (high: 250 pg), were injected, using a Sutter Instruments microinjector, into separate batches of embryos (n = 100–120) from heterozygous *rbpr2* ^musc97^ parents, at the 1–2 cell development stage. At 5 dpf, twenty randomly selected injected larvae were imaged and then individually genotyped by direct sequencing as outlined above. Rescue experiments were repeated twice, with a new preparation of capped and polyadenylated *rbpr2* mRNA. Histological analysis of homozygous *rbpr2* ^musc97^ mutant larvae eyes after rescue experiments were performed at 5 dpf.

### *Rbpr2*^musc97^ mutant zebrafish all-*trans* Retinoic Acid rescue experiments

Exogenous applied all-*trans* RA (Sigma) was dissolved in DMSO and applied at two different does (0.1 μM and 0.2 μM) to the fish water containing embryos (from heterozygous *rbpr2* ^musc97^ parents) at the 40% epiboly stage, just before gastrulation begins. Controls embryos were incubated with the vehicle only (0.1% DMSO) showed no rescue of phenotype. Twenty-four larvae were imaged at 5 dpf and genotyped. Experiments were repeated twice. Histological analysis of homozygous *rbpr2* ^musc97^ mutant larvae eyes after rescue experiments were performed at 5 dpf.

### Transmission Electron microscopy (TEM)

Control/WT and *rbpr2* ^musc97^ mutant larvae were fixed in a solution containing 2.5% glutaraldehyde, 2% paraformaldehyde, and postfixed with 2% osmium tetroxide. The fixed tissue was sectioned to obtain radial sections at 1 μm and rinsed with cacodylate buffer (0.1 M), dehydrated through a graded ethanol series, and infiltrated with Epon resin. Samples were processed by the Electron Microscopy Resource Laboratory at the Medical University of South Carolina using a Joel Transmission Electron Microscope (JEM-1400Plus)^58^.

### Western Blot Analysis

Total protein from cells was isolated using the M-PER protein lysis buffer (ThermoScientific, Beverly, MA) containing protease inhibitors (Roche, Indianapolis, IN). Approximately 25 μg of total protein was electrophoresed on 4–12% SDS-PAGE gels and then transferred to PVDF membranes^34^. Membranes were then probed with primary antibodies against β-actin (1:10,000, Sigma), anti-LRAT (1:500, Abcam) and anti-V5 (1:1000, Sigma) in antibody buffer (0.2% Triton X-100, 2% BSA, 1X PBS). HRP conjugated secondary antibodies (BioRad, Hercules, CA) were used at 1:10,000 dilution. Protein expression was detected by autoradiography and relative intensities of each band were quantified (densitometry) using ImageJ Software version 1.49, and normalized to the loading control.

### Quantitative Real Time-PCR

RNA was obtained from heads of 5.5 dpf *rbpr2* ^*musc97*^ mutant (n = 12) and WT larvae (n = 15), and isolated using Trizol reagent, and processed as described previously^18^. One microgram of total RNA was reverse transcribed using the SuperScript II cDNA Synthesis Kit (Invitrogen, Eugene, OR). Quantitative Real-Time PCR (qRT-PCR) was carried out using the SYBR green 1 chemistry (BioRad, Hercules, CA) and gene specific primers pairs for zebrafish *rbpr2* (forward 5′-TCAGACTGAGAGTGTGTTTAC-3′ and reverse 5′-TACTGGCGGTGGTTTCATGACCT-3′), zebrafish *aldh1a1* (forward 5′-TTCAACGTAGACTATGTAGAAAA-3′ and reverse 5′-AGCGACTGCTTTTTCCACA-3′), zebrafish *aldh1a2* (forward 5′-CATTTTTGCAGATGCTGA TTT TG-3′ and reverse 5′- CAAAGATACGGGAACCAGCAGT-3′), zebrafish *cyp26a1* (forward 5′-ATAAAGACGGACGAGCAAGA-3′ and reverse 5-TCGTCATCTTGAATTTTCTT-3′), zebrafish *lrat* (forward 5′-CGCGTACGGAGCTCCGATTC-3′ and reverse 5′-AACTCACCTTGTCGGTCTGC-3′), zebrafish *dhrs3a* (forward 5′- GTCGGGGATGTCACCATTCTT-3′ and reverse 5′- ATTTGTCTCTTACCCAGAACT-3′), zebrafish *rpe65* (forward 5′- GTTTTTCTCATATTTTAAGGG-3′ and reverse 5′- CTTTTTTAGCGTTTCCAGAGTG-3′) zebrafish rbp4 (forward 5′-ACAACATTGTGGCCAATTTCAAA-3′ and reverse 5′-CGCAGCAGCTCCCCAGTACTT-3′). Retina gene expression was normalized to 18 S ribosomal RNA expression (forward 5′-TCGCTAGTTGGCATCGTTTATG-3′ and reverse 5′- CGGAGGTTCGAAGACGATCA-3′). Samples for qRT-PCR experiments were assayed in triplicate using the BioRad CFX96 qRT-PCR machine. Each experiment was repeated twice, using newly synthesized cDNA. The ΔΔCt method was employed to calculate fold changes^34^.

### Optokinetic Response (OKR)

Optokinetic response measurements were conducted on 5.5 dpf zebrafish larvae using the VisioTracker system (VisioTracker 302060 Series; TSE Systems GmbH, Bad Homburg, Germany). Contrast sensitivity was assessed as described previously^32^. For the spatial frequency response function, the contrast was held constant at 70% and we tested stimuli of 0.011, 0.025, 0.05 and 0.10 cycles/deg by first increasing and then decreasing the frequency. Each spatial frequency stimulus was presented for 3 seconds before reversing direction for another 3 seconds to minimize saccade frequency. All OKR stimuli were presented with a constant angular velocity of 7.5° per second. The genotypes of individual larvae were confirmed sequencing following OKR tests.

### TUNEL Assay

Frozen sections from retinas were stained using an *in-situ* fluorescein cell death detection kit, (Roche, Mannheim, Germany) according to the manufacturer′s instructions^34^. Sections were photographed using confocal microscopy (SP8, Leica, Germany).

### Statistical Analyses

Results are presented as mean±s.d. and the number of experiments is indicated in the figure legends. Statistical significance was assessed using the two-tailed Student’s *t*-test. For western blot analysis, relative intensities of each band were quantified (densitometry) using the ImageJ Software version 1.49 and normalized to the loading control β-Actin. QRT-PCR analysis was normalized to 18 S RNA, and the ΔΔCt method was employed to calculate fold changes. Statistical significance was assessed by using the two-tailed Student’s *t*-test.

## Electronic supplementary material

Supplementary Information
