# Supplementary material for: The Retinol Binding Protein Receptor 2 (Rbpr2) is required for Photoreceptor Outer Segment Morphogenesis and Visual Function in Zebrafish
Source: Sci Rep. 2017 Nov 24;7:16207. doi: 10.1038/s41598-017-16498-9 (PMC5701214; doi:10.1038/s41598-017-16498-9)

Supplementary Information

The Retinol Binding Protein Receptor 2 (Rbpr2) is required for Photoreceptor Outer Segment Morphogenesis and Visual Function in Zebrafish

Authors: Yi Shi[1,2], Elisabeth Obert[3], Bushra Rahman[1], Bärbel Rohrer[3,4] and Glenn P. Lobo[1,3*]

Affiliations: [1]Department of Medicine, Medical University of South Carolina, Charleston, SC, 29425, USA. [2]Tianjin Medical University Eye Hospital, Tianjin, 300384, China. [3]Department of Ophthalmology, Medical University of South Carolina, Charleston, SC, 29425, USA. [4]Ralph H. Johnson VA Medical Center, Division of Research, Charleston, SC 2940, USA.

***Corresponding Author**

Glenn P. Lobo, Ph.D.

Email: lobo@musc.edu

Supplementary Information

Supplementary Figure S1: Rbpr2 mRNA expression patterns in staged zebrafish larvae

(**A**) Rbpr2 expression patterns were determined in staged zebrafish larvae by semi-quantitative RT-PCR. Total RNA from zebrafish larvae (1 - 5.5 dpf.) were subjected to semi-quantitative RT-PCR using zebrafish *rbpr2, stra6* and *gapdh* gene specific primers. Amplified PCR products were electrophoresed on 2 % agarose gels, alongside a low-molecular weight DNA ladder. Zebrafish *rbpR2* mRNA was observed as being expressed from early to late larvae stages. A portion of Zebrafish *stra6* was amplified and used as positive control. A portion of zebrafish *gapdh* was also amplified at same developmental time points and this served as the endogenous loading control.

Supplementary Figure S2: Eye sections of zebrafish at 5 dpf from rescue experiments

Histological analysis of *rbpr2*^{musc97} mutant larvae eyes after rescue experiments at 5 dpf showed normal retinal lamination and eye patterning which were comparable to WT animals.

Supplementary Figure S3: ZIRC *rbpr2* mutants (*rbpr2*^{sa10706}) show eye phenotypes consistent with retinol deficiency

A *rbpr2* mutant zebrafish line (G>A mutation; *rbpr2*^{sa10706}) from the Zebrafish International Resource Center (ZIRC) which affects the essential splice site of exon

5/intron 6, was obtained and analyzed by light microscopy, histology and immunohistochemistry for cones at 5.5 dpf. The resulting eye phenotype observed was similar to the TALEN generated *rbpr2* mutant phenotype (*rbpr2*^{musc97}) described. *Rbpr2*^{sa10706} mutants showed gross defects, which included: *hydrocephaly; **smaller eyes, ***pericardial edema and ****slight tail curvature.

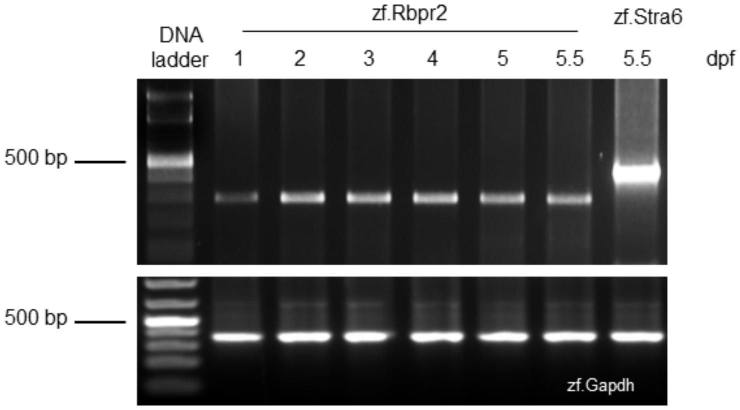

Semi-quantitative PCR at 25 cycles

Supplementary Figure 1

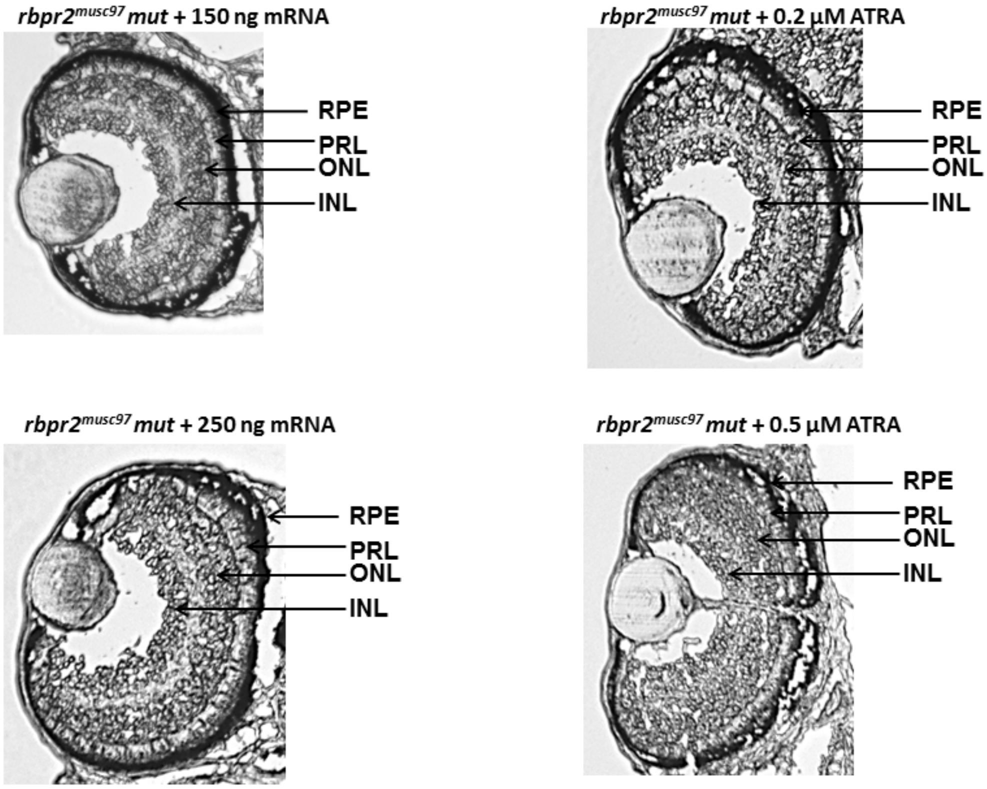

Supplementary Figure 2

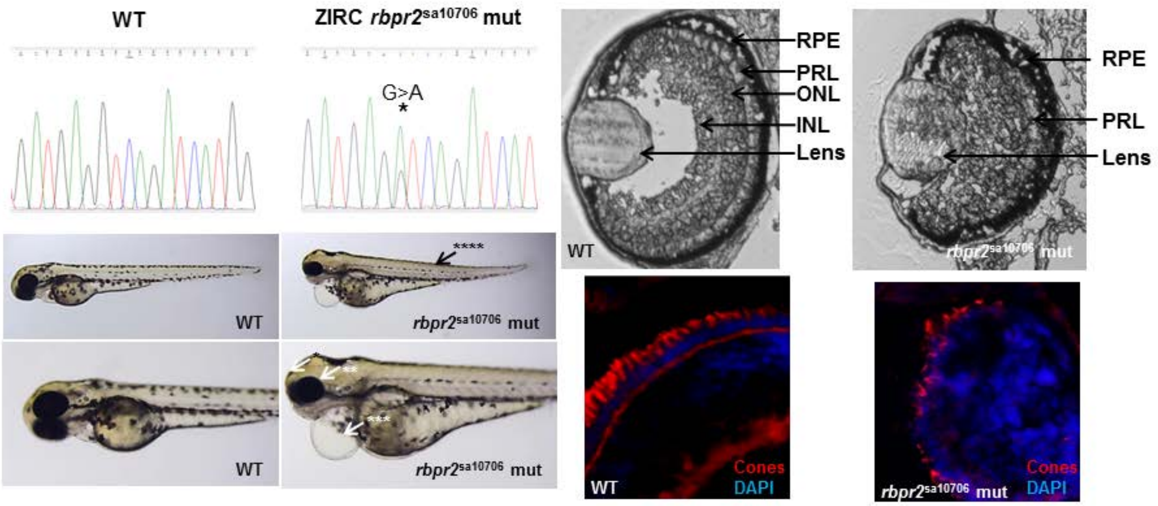

Supplementary Figure 3

Full image of unedited gel showing expression of V5-tagged Rbpr2- (V5 antibody) and LRAT in stable cell lines: Supporting Figure 1A

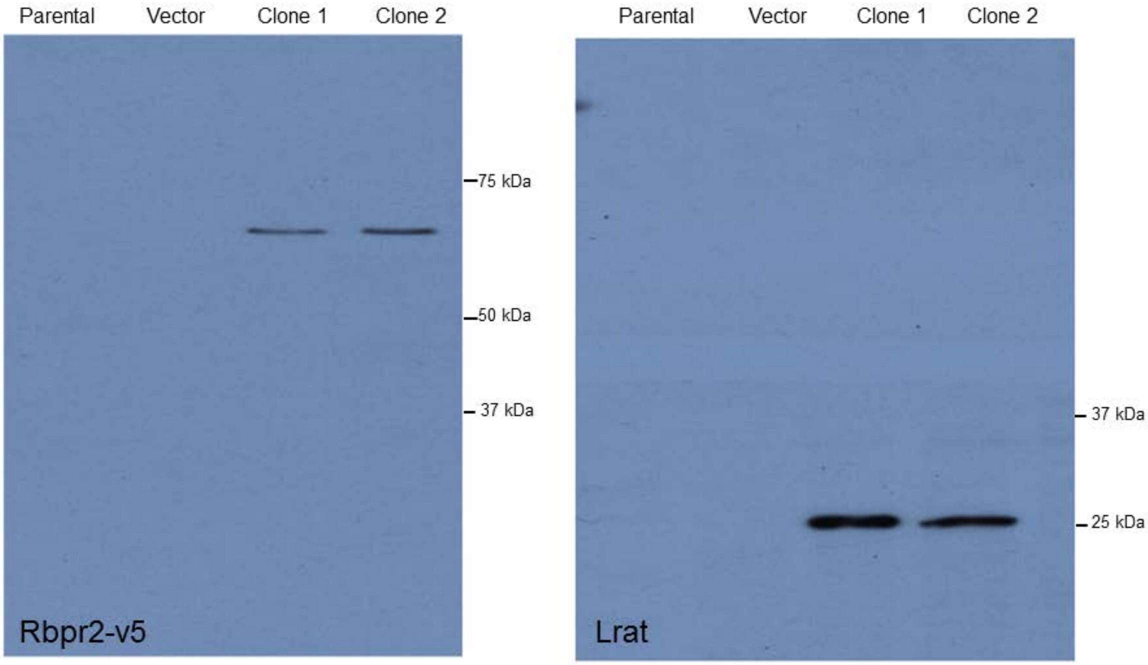

Full image of unedited gel showing expression of β-actin in stable cell lines: Supporting Figure 1A

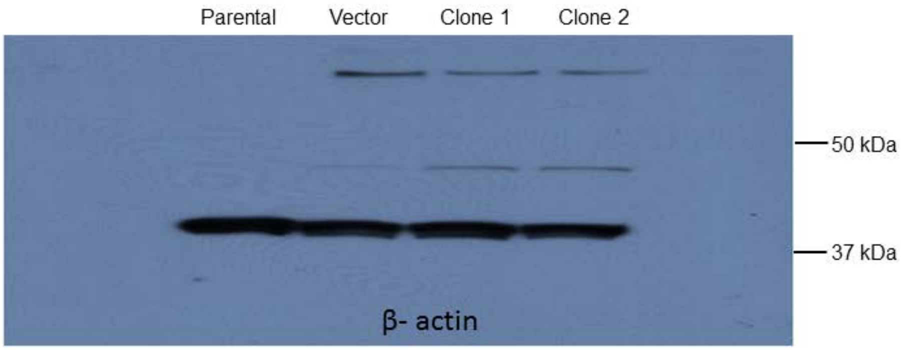

Full image of SDS-PAGE gel and Commassie Blue staining showing expression of purified RBP4 protein: Supporting Figure 1D

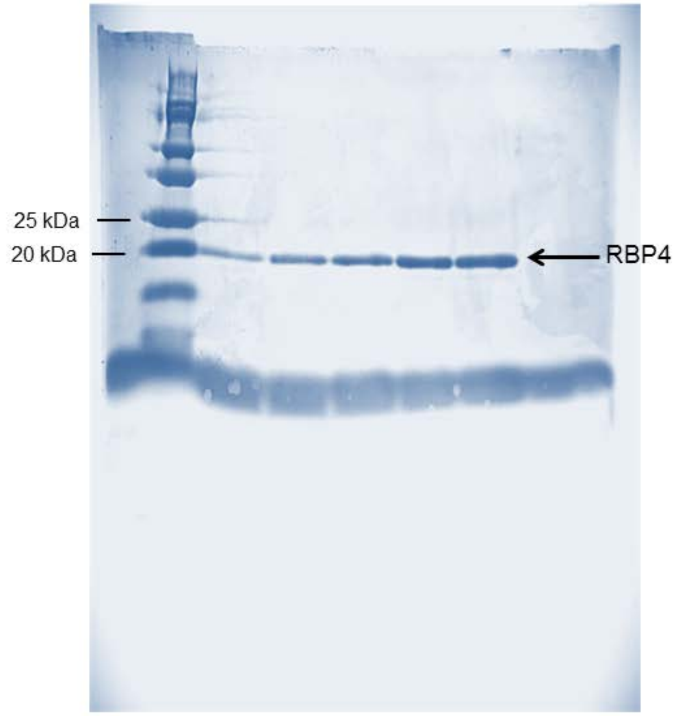

Supplement: Supplementary file 1 — Supplementary Information [file 41598_2017_16498_MOESM1_ESM.pdf]
